# Pelvic lymph-node staging with ^18^F-DCFPyL PET/CT prior to extended pelvic lymph-node dissection in primary prostate cancer - the SALT trial -

**DOI:** 10.1007/s00259-020-04974-w

**Published:** 2020-08-12

**Authors:** B. H. E. Jansen, Y. J. L. Bodar, G. J. C. Zwezerijnen, D. Meijer, J. P. van der Voorn, J. A. Nieuwenhuijzen, M. Wondergem, T. A. Roeleveld, R. Boellaard, O. S. Hoekstra, R. J. A. van Moorselaar, D. E. Oprea-Lager, A. N. Vis

**Affiliations:** 1 Department of Urology, Amsterdam University Medical Centres (VU University), De Boelelaan 1117, 1081 HV Amsterdam, The Netherlands; 2 Department of Radiology & Nuclear medicine, Amsterdam University Medical Centres (VU University), De Boelelaan 1117, 1081 HV Amsterdam, The Netherlands; 3 Prostate Cancer Network, De Boelelaan 1117, 1081 HV Amsterdam, The Netherlands; 4 Department of Pathology, Amsterdam University Medical Centres (VU University), Amsterdam, The Netherlands; 5 Department of Nuclear medicine, Noordwest Ziekenhuisgroep, Alkmaar, The Netherlands; 6 Department of Urology, Noordwest Ziekenhuisgroep, Alkmaar, The Netherlands

**Keywords:** Prostate cancer, PSMA-ligand, Primary staging, ^18^F-DCFPyL PET/CT, Lymph-node metastasis

## Abstract

**Purpose:**

The detection of lymph-node metastases (N1) with conventional imaging such as magnetic resonance imaging (MRI) and computed tomography (CT) is inadequate for primarily diagnosed prostate cancer (PCa). Prostate-specific membrane antigen (PSMA) PET/CT is successfully introduced for the staging of (biochemically) recurrent PCa. Besides the frequently used ^68^gallium-labelled PSMA tracers, ^18^fluorine-labelled PSMA tracers are available. This study examined the diagnostic accuracy of ^18^F-DCFPyL (PSMA) PET/CT for lymph-node staging in primary PCa.

**Methods:**

This was a prospective, multicentre cohort study. Patients with primary PCa underwent ^18^F-DCFPyL PET/CT prior to robot-assisted radical prostatectomy (RARP) with extended pelvic lymph-node dissection (ePLND). Patients were included between October 2017 and January 2020. A Memorial Sloan Kettering Cancer Centre (MSKCC) nomogram risk probability of ≥ 8% of lymph-node metastases was set to perform ePLND. All images were reviewed by two experienced nuclear physicians, and were compared with post-operative histopathologic results.

**Results:**

A total of 117 patients was analysed. Lymph-node metastases (N1) were histologically diagnosed in 17/117 patients (14.5%). The sensitivity, specificity, positive predictive value and negative predictive value for the ^18^F-DCFPyL PET/CT detection of pelvic lymph-node metastases on a patient level were 41.2% (confidence interval (CI): 19.4–66.5%), 94.0% (CI 86.9–97.5%), 53.8% (CI 26.1–79.6%) and 90.4% (CI 82.6–95.0%), respectively.

**Conclusion:**

^18^F-DCFPyL PET/CT showed a high specificity (94.4%), yet a limited sensitivity (41.2%) for the detection of pelvic lymph-node metastases in primary PCa. This implies that current PSMA PET/CT imaging cannot replace diagnostic ePLND. Further research is necessary to define the exact place of PSMA PET/CT imaging in the primary staging of PCa.

## Introduction

Prostate cancer (PCa) is the most frequently diagnosed cancer in men in the Western world [1, 2]. Initial therapy includes robot-assisted radical prostatectomy (RARP) and external beam radiation therapy. Accurate assessment of local tumour stage (T-stage), regional lymph-node involvement (N-stage) and screening for distant metastases (M-stage) is essential, since it significantly affects patient follow-up, therapeutic decisions and oncological outcome [3]. Conventional imaging studies such as computed tomography (CT) and magnetic resonance imaging (MRI) have moderate sensitivity for the detection of lymph-node metastases (42% and 39%, respectively) [4–6]. Therefore, extended pelvic lymph-node dissection (ePLND) remains the preferred technique for nodal staging. It is an invasive procedure, however, associated with complications such as lymphocele, deep venous thrombosis, and longer hospital stay [7].

Recently, radiolabelled prostate-specific membrane antigen (PSMA) has been introduced. PSMA is a class-II trans-membrane glycoprotein that provides a valuable target for radiolabelled imaging as it is significantly overexpressed in malignant prostate cells [8]. Moreover, its expression is associated with tumour grade, stage and the occurrence of metastases [9, 10]. So far, most experience has been obtained in patients with biochemically recurrent (BCR) PCa after initial curative local therapy, and using ^68^gallium-labelled PSMA tracers. High detection rates for metastases were demonstrated even at low prostate-specific antigen (PSA) values (i.e. 45% for PSA < 0.5 ng/mL and over 95% for PSA ≥ 2.0 ng/mL) [11]. Alternatively, ^18^fluorine-labelled PSMA tracers have been developed, such as ^18^F-DCFPyL (2-(3-(1-carboxy-5-[(6-[18F]fluoro-pyridine-3-carbonyl)-amino]-pentyl)-ureido)-pentanedioic acid) [12, 13]. Due to a shorter positron range and higher positron yield, the ^18^F-radionuclide provides a higher PET-image resolution compared with ^68^Ga, which may improve detection of small (lymph-node) metastases. Somewhat higher detection rates were observed for ^18^F-DCFPyL, as compared with ^68^Ga-PSMA, in patients with BCR undergoing consecutive scans with both tracers [14, 15].

Only few studies have evaluated the accuracy of PSMA PET/CT for nodal staging of primary PCa, comparing scan results with the histopathology of surgical ePLND specimens. In patients with intermediate and high-risk disease, ^68^Ga-PSMA showed modest sensitivity for lymph-node metastatic disease, at consistently high specificity [16–19]. For ^18^F-DCFPyL PET/CT, only a single small prospective series for staging newly diagnosed PCa has been published yet [20].

This is the first large, prospective study on the staging accuracy for the detection of PCa lymph-node metastases using ^18^F-DCFPyL PET/CT imaging (acronym: SALT trial). The primary aim was to assess the accuracy to detect pelvic lymph-node metastases with ^18^F-DCFPyL PET/CT, in patients with intermediate and high-risk PCa. As a secondary objective, the ability of ^18^F-DCFPyL PET/CT to predict the pathological local tumour stage (pT) was assessed. The imaging results from ^18^F-DCFPyL PET/CT were compared with final histopathology from the ePLND and radical prostatectomy.

## Methods

### Study design and patient population

This was a prospective, non-randomized study evaluating the accuracy of ^18^F-DCFPyL PET/CT for detecting pelvic lymph-node metastases in patients with primary PCa undergoing radical surgery. Imaging results were compared with histopathology following RARP and ePLND (reference standard). The study has been approved by the ethical review board of the Amsterdam University Medical Centre (review number 2017.543), and was registered in the Netherlands Trial Register (NTR 6754). All subjects were included consecutively, and signed informed consent for collection of their clinical data and analysis of the ^18^F-DCFPyL PET/CT scans, prior to RARP. Patients were enrolled between October 2017 and January 2020 in two reference centres of the Prostate Cancer Network Netherlands (Amsterdam University Medical Centre; Noordwest ziekenhuisgroep). Inclusion criteria were (1) histologically proven, intermediate or high-risk PCa [3] and (2) patients undergoing RARP and ePLND. Patients with distant metastases were not considered for evaluation, as no RARP and ePLND is performed in such cases. Of all included patients, age, prostate volume, initial PSA level, pathological biopsy features (histopathological grade, number of cores with cancer) and EAU risk category were collected [3]. The indication to perform ePLND was based on a ≥ 8% risk of lymph-node involvement as predicted by the Memorial Sloan Kettering Cancer Centre (MSKCC) nomogram [21], or on the presence of high-risk features: PSA > 20 ng/mL, Gleason score 8–10 or suspicion of cT2c or higher [3]. Patients with incomplete ePLND due to intraoperative difficulty in performing ePLND were excluded from final analysis. The required sample size was calculated at 120 patients, and was based on a 30% incidence of lymph-node metastases overall, with an estimated sensitivity of 90% (lower boundary of the 95% confidence interval (CI) at 80%) [22, 23].

### Image acquisition

Patients were staged with ^18^F-DCFPyL PET/CT in the Amsterdam UMC or Noordwest ziekenhuisgroep. ^18^F-DCFPyL was synthesized under good manufacturing practices conditions at both centres. PET images were made at a median of 118 min after injection (interquartile range [IQR] 112–123 min) of a median dose of 311 MBq ^18^F-DCFPyL (IQR 297–324 MBq) within a median of 4.1 weeks (IQR 2.1–6.6) after prostate biopsy and within a median of 5.9 weeks (IQR 3.6–12.0) prior to surgery. Image acquisitions were performed using a Philips Ingenuity TF (Philips Healthcare®, the Netherlands/USA) and a Siemens Biograph-16 TruePoint (Siemens Healthineers®, Germany) PET/CT system. The scan trajectory included mid-thigh to skull base, with 4 min (Philips) and 5 min (Siemens) per bed position. All PET scans were combined with a low-dose CT (33/117 patients) or contrast-enhanced CT scan (84/117 patients) (30–110 mAs, 110–130 kV). Images were corrected for decay, scatter, random coincidences and photon attenuation.

Images were reconstructed with a BLOB-based Ordered-Subsets Expectations Maximization algorithm (Philips, 3 iterations; 33 subsets) [24] and the Ordered-Subsets Expectations Maximization algorithm (Siemens, 4 iterations; 16 subsets, including a 5 mm Gaussian filter). The reconstructed images had a matrix size 288 × 288 with voxel size 2 × 2 × 2 mm (Philips) and a maximum matrix size of 256 × 256 and smallest voxel size 2.67 × 2.67 × 4mm (Siemens).

### Scan interpretation

All scans were clinically and prospectively interpreted in the participating centres by one of two nuclear medicine physicians (DO, MW) with ample experience in PSMA PET interpretation (> 300 scans). Upon completion of the study, all scans were reviewed by a second independent reader who was blinded to initial scan interpretation, surgery and histopathology results (DO, GZ). A joint re-evaluation was performed in case of incongruent scan interpretation (consensus read), and used for final analysis. Lymph-node metastases were defined as increased PSMA expression, higher than the background, incompatible with physiological uptake, and in a typical site of PCa. A significant CT substrate was not an absolute prerequisite. The following parameters were recorded: detection of the primary tumour, tumour stage and presence of pelvic lymph-node metastases (N1). Pelvic lymph-node metastases were further classified in accordance with the four sections of the ePLND (external iliac artery left/right; obturator fossa left/right).

### Inter-observer variability

To assess inter-observer variability, the proportional agreement was calculated, based on the two individual scan interpretations [25]. Proportional agreement was defined as the degree to which the two independent PET/CT readings were reported as the same. Positive agreement was defined as the degree to which the two independent PET/CT readings were reported both positively, whereas negative agreement was defined as the degree to which the two independent PET/CT readings were reported both negatively.

### Semi-quantitative analysis

PET/CT scans with PSMA-avid lesions in the prostate were delineated according to the reports of the nuclear medicine physicians. Semi-automatic delineation on the PET scans from both participating centres was performed with the in-house developed ACCURATE tool© [26]. The ACCURATE tool semi-automatically generated a volume-of-interest using a 50% isocontour based on standard uptake value peak (SUV_peak_), with correction for background uptake [26]. SUV_peak_ is defined as the highest local intensity of uptake with a 6-mm-radius sphere [27]. To compare the median SUV_peak_ of the prostate tumour for patients with and without lymph-node metastases, the Mann-Whitney *U* test was used (significance set at *p* < 0.05). To compare the median SUV_peak_ of the prostate tumour with the corresponding Gleason score of the lesion, the Kruskall-Wallis test was used (significance set at *p* < 0.05). A linear regression was run to predict PSA from SUV_peak_ of the prostate index lesion (significance set at *p* < 0.05).

### Surgical procedure and histopathologic evaluation

The ePLND surgical template includes removal of fatty lymphoid tissue overlying the common and external iliac vessels and within the obturator fossa [3]. The medial border of the surgical template of ePLND concerns the ureter, the caudal border the obturator nerve and the cranial border the genitofemoral nerve continued to the inner aspect of the pubic bone. All four template sections were individually collected and presented for histopathologic analysis.

### Histopathology of resected specimens

Prostate specimens and resected lymph-node templates were fixated in formaldehyde (10%) directly after surgery and processed according to routine clinical standards [3]. Individual lymph nodes were manually picked from the surgical specimens and sectioned (< 3 mm as a whole, 3–10 mm in half and > 10 mm in multiple) to make histologic slices. All slices were reviewed by dedicated uro-pathologists (unblinded from PET results), reporting tumour Gleason score, pathological tumour stage (pT) and total number of resected lymph nodes. The maximum diameter (mm) of metastatic nodal deposits was recorded (mm).

### Outcome measurement and statistical analysis

The primary outcome of this study was the patient-based sensitivity of ^18^F-DCFPyL PET/CT to detect pelvic lymph-node metastases. The diagnostic accuracy of ^18^F-DCFPyL PET/CT was calculated with histopathologic evaluation of ePLND as a reference. The sensitivity, specificity and positive and negative predictive values (PPV and NPV, respectively) of ^18^F-DCFPyL PET/CT for the detection of pelvic lymph-node metastases (pN1) were calculated both on a patient level and on a surgical template level. The surgical template analysis was based on the abovementioned 4 surgical templates of the ePLND, and was applied to approximate lesion based-detection using ^18^F-DCFPyL PET/CT. To compare the median diameter of PET/CT-detected lymph-node metastases vs. PET/CT-undetected lymph-node metastases, the Mann-Whitney *U* test was used (significance set at *p* < 0.05).

For the assessment for the local tumour stage (pT), we measured the accuracy of ^18^F-DCFPyL PET/CT to differentiate local advancement (T3 a-b, T4) from prostate-confined disease (T2). This study did not investigate the exact location of local PCa advancement, only the presence of extracapsular or seminal vesicle invasion was noted. Local advancement was defined as PSMA expression outside the borders of the prostate gland, not suspect for overprojection or bladder/urethral physiological activity. Numerical variables were summarized with median values and interquartile ranges (IQR), categorical variables with proportions (%). Statistical analysis was done with IBM® SPSS® Statistics for Windows®, version 26.

## Results

### Patient characteristics

**Fig. 1:**
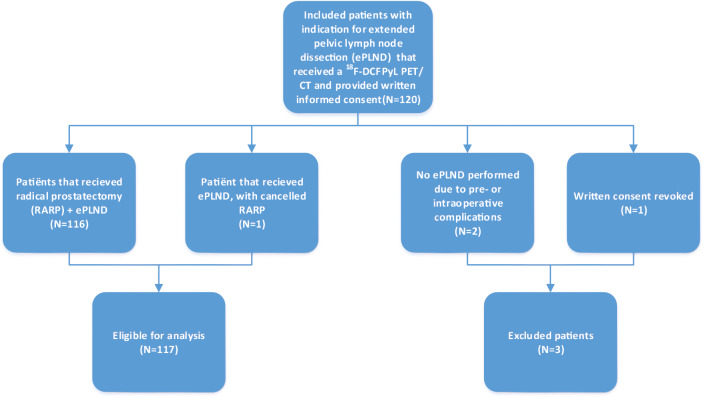
Study flowchart

A total of 120 patients were included in this study, and scheduled for ePLND with RALP after ^18^F-DCFPyL PET/CT, as presented in Fig. 1. Three patients were excluded from the final analysis: one patient ultimately proved to be unfit for surgery, and the second was excluded because surgery was not completed due to intraoperative complications (intestinal perforation). A third patient revoked his informed consent during the study period, after initial consent. One patient did not receive a complete prostate removal due to persistent intraoperative bleeding, which made the surgeon decide not to continue surgical resection of the prostate. The preceding ePLND was completed, however, and the patient was included for analysis of lymph-node metastases. Therefore, a total of 117 patients were included for final analysis for the accuracy of N-staging, and 116 patients for the accuracy of T-staging. Included patients had a median age of 67 years (IQR 61–70), and a median initial PSA level of 10.9 ng/mL (IQR 7.2–20.8). According to EAU guidelines, 43/117 (36.8%) patients had intermediate risk PCa and 74/117 (63.3%) had high-risk PCa [3]. The median MSKCC risk for lymph-node metastases was 14.3% (IQR 10.1–30.2). Preoperative characteristics of included patients are listed in Table 1.

**Table 1:** Preoperative characteristics of patients undergoing ^18^F-DCFPyL PET/CT before robot-assisted radical prostatectomy and extended pelvic lymph-node dissection

Baseline characteristics			
		Median	IQR
Age (years)		67	61–70
Prostate volume (mL)		39	30–56
Initial PSA (ng/mL)		10.9	7.2–20.8
Positive biopsy cores (% of total cores)		50	36.6–73.9
MSKCC risk of lymph-node metastases (%)		14.3	10.1–30.2
		*n*	%
Biopsy ISUP category [3]^a^	1	5	4.3
	2	37	31.6
	3	26	22.2
	4	31	26.5
	5	18	15.4
	Total	117	100.0
Clinical T-stage	1c	44	37.6
	2a/b	54	46.2
	2c	11	9.4
	3a	7	6.0
	Missing	1	0.9
	Total	117	100.0
EAU risk category [3]	Intermediate	43	36.8
	High	74	63.3
	Total	117	100.0

*IQR*, interquartile range; *PSA*, prostate-specific antigen; *MSKCC*, Memorial Sloan Kettering Cancer Centre; *ISUP*, International Society of Urological Pathology; *EAU*, European Association of Urology

^a^ISUP definition

ISUP 1 = Gleason score 3 + 3 = 6

ISUP 2 = Gleason score 3 + 4 = 7

ISUP 3 = Gleason score 4 + 3 = 7

ISUP 4 = Gleason score 4 + 4 = 8/Gleason score 3 + 5 = 8/Gleason score 5 + 3 = 8

ISUP 5 = Gleason score 4 + 5 = 9/Gleason score 5 + 4 = 9/Gleason score 5 + 5 = 10

### Accuracy of ^18^F-DCFPyL PET/CT for detecting pelvic lymph-node metastases

Pathological features after RARP and ePLND are listed in Table 2. A total of 2149 lymph nodes were resected during surgery (median 18 lymph nodes per patient, IQR 13–23). In 17/117 patients, lymph-node metastases were diagnosed (14.5% of total). Of the 17 patients with lymph-node metastases on histopathological evaluation, 7 patients had a ^18^F-DCFPyL PET/CT suspicious for lymph-node metastases. Hence, the patient-based sensitivity to detect lymph-node metastases using ^18^F-DCFPyL PET/CT was 41.2% (95% confidence interval (CI) 19.4–66.5), with a specificity of 94.0% (95%CI 86.9–97.5), a PPV of 53.8% (95%CI 26.1–79.6) and a NPV of 90.4% (95%CI 82.6–95.0), as shown in Table 3.

**Table 2:** Post-operative histopathologic features of patients who underwent a robot-assisted radical prostatectomy and extended pelvic lymph-node dissection

Pathology results		*n*	%
ISUP category [3]^a^	1	1	0.9
	2	46	39.3
	3	39	33.3
	4	7	5.9
	5	23	20.8
	n.a.^b^	1	0.9
	Total	117	100.0
Pathological tumour stage (pT)-stage	pT2	54	46.2
	pT3a	44	37.6
	pT3b	17	14.5
	pT4a	1	0.9
	n.a.^b^	1	0.9
	Total	117	100.0
Lymph-node (N)-stage	0	100	85.4
	1	17	14.5
	Total	117	100.0
		*n* (nodes)	*%*
Dissected lymph nodes	Benign	2118	98.6
	Malign	31	1.4
	Total	2149	100.0

*n.a.*, not available; *ISUP*, International Society of Urological Pathology

^a^ISUP definition

ISUP 1 = Gleason score 3 + 3 = 6

ISUP 2 = Gleason score 3 + 4 = 7

ISUP 3 = Gleason score 4 + 3 = 7

ISUP 4 = Gleason score 4 + 4 = 8/Gleason score 3 + 5 = 8/Gleason score 5 + 3 = 8

ISUP 5 = Gleason score 4 + 5 = 9/Gleason score 5 + 4 = 9/Gleason score 5 + 5 = 10

^b^In one patient, extended lymph-node dissection was successfully performed, yet surgical removal of the prostate proved unfeasible due to extensive intraoperative bleeding

**Table 3:** The diagnostic value of ^18^F-DCFPyL PET/CT for detecting lymph-node metastatic disease on a per-patient and template basis

Patient-based accuracy					
	pN1	pN0	Total	% (95%CI)	
cN1	7	6	13	53.8 (26.1–79.6)	PPV
cN0	10	94	104	90.4 (82.6–95.0)	NPV
Total	17	100	117	14.5	Prevalence
% (95%CI)	41.2 (19.4–66.5)	94.0 (86.9–97.5)			
	Sensitivity	Specificity			
Template-based accuracy					
	pN1	pN0	Total	% (95%CI)	
cN1	8	10	18	44.4 (22.4–68.6)	PPV
cN0	15	435	450	96.6 (94.4–98.0)	NPV
Total	23	445	468	4.9	Prevalence
% (95%CI)	34.7 (17.1–57.1)	97.7 (95.7–98.9)			
	Sensitivity	Specificity			

*CI*, confidence interval

**Fig. 2:**
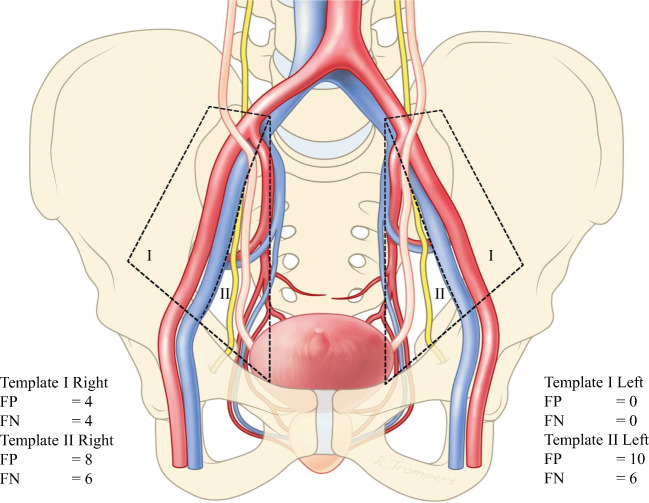
Schematic overview of the false-positive and false-negative lymph-node findings when comparing ^18^F-DCFPyL PET/CT with histopathology, classified according to the templates used in the extended pelvic lymph-node dissection. Template I (green) involves lymph nodes surrounding the arteria iliaca externa. Template II (red) involves lymph nodes surrounding the arteria iliaca interna, and the nervus obturatorius. FP, false-positive lymph-node finding; FN, false-negative lymph-node finding

In the 17 patients with lymph-node metastases, 31 lymph-node metastases were histologically identified in 23 surgical ePLND templates. ^18^F-DCFPyL PET/CT preoperatively identified 38 PSMA-avid regions suspect for lymph-node metastases in 18 surgical ePLND templates. Therefore, the template-based sensitivity for the detection of lymph-node metastases using ^18^F-DCFPyL PET/CT was 34.7% (95%CI 17.1–57.1), with a specificity of 97.7% (95%CI 95.7–98.9), a PPV of 44.4 (95%CI 22.4–68.6) and a NPV of 96.6% (95%CI 94.4–98.0) as seen in Table 3. For a detailed overview of false-positive and negative lymph nodes that were misdiagnosed by ^18^F-DCFPyL PET/CT, please see Fig. 2.

**Fig. 3:**
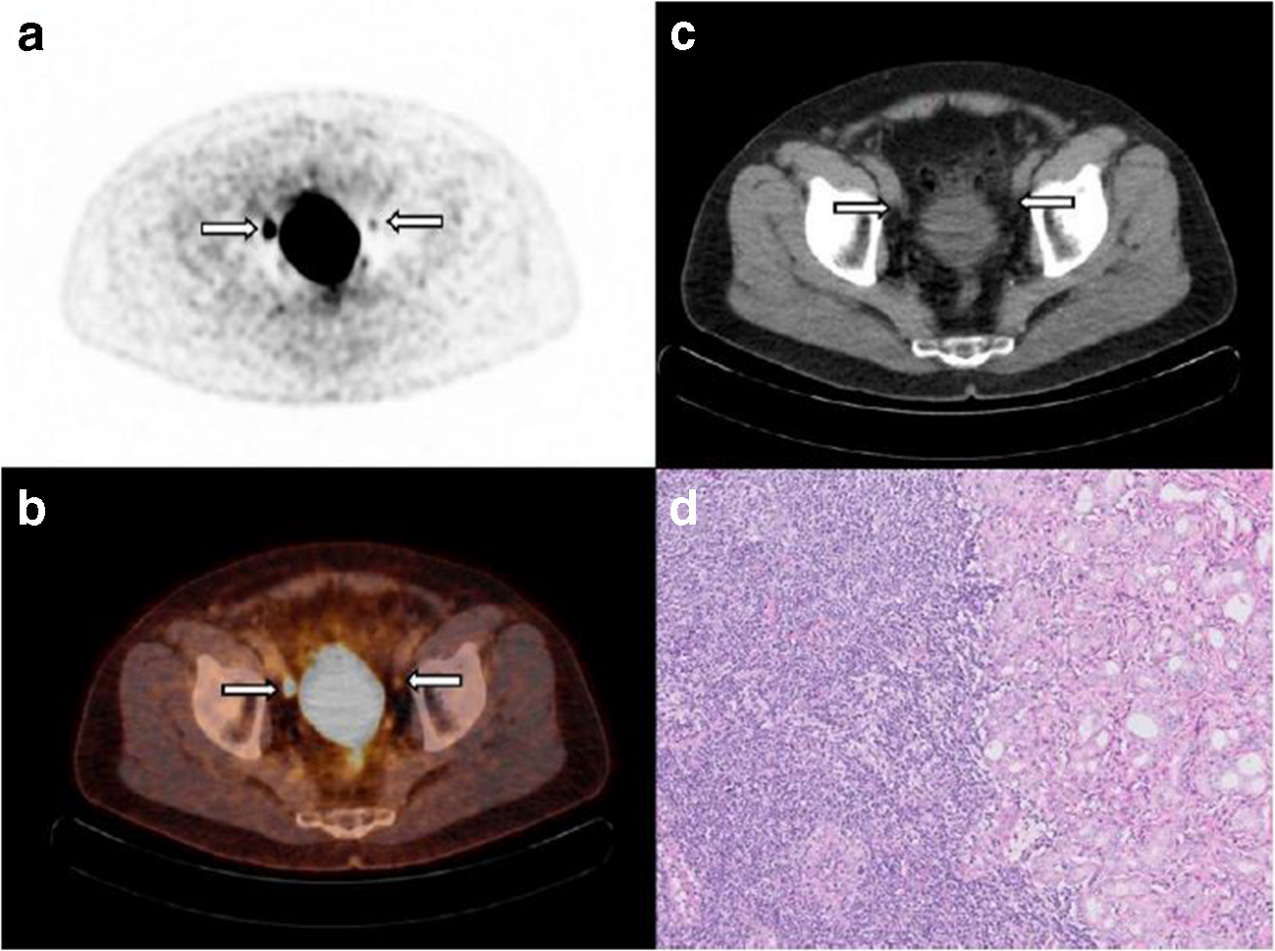
A 68-year-old man with cT2c, Gleason score 3 + 4 = 7 prostate cancer and initial PSA 10.4 ng/mL considered candidate for radical prostatectomy with extended pelvic lymph-node dissection. MSKCC nomogram showed 10.8% risk of lymph-node involvement. Transversal ^18^F-DCFPyL PET (**a**) and fused PET/CT (**b**) show intense uptake in the pelvic region right, corresponding with an enlarged 10-mm lymph node adjacent to the right external iliac artery on CT (**c**), suspect for lymph-node metastasis (**a**–**c**, left arrow). A contralateral focus with faint uptake is observed on PET and fused PET/CT in the pelvic region, without an evident morphologic substrate on CT. Due to the minimal tracer uptake (above the blood pool and lower than the liver); this left-sided focus was not suspect for lymph-node metastasis after dual reading. After surgical resection of 26 lymph nodes, post-operative histopathology revealed a right-sided right iliac lymph-node metastasis measuring 10 mm, as well as a left iliac lymph-node metastasis of 5 mm, haematoxylin and eosin stain, original magnification × 10 (**d**)

**Fig. 4:**
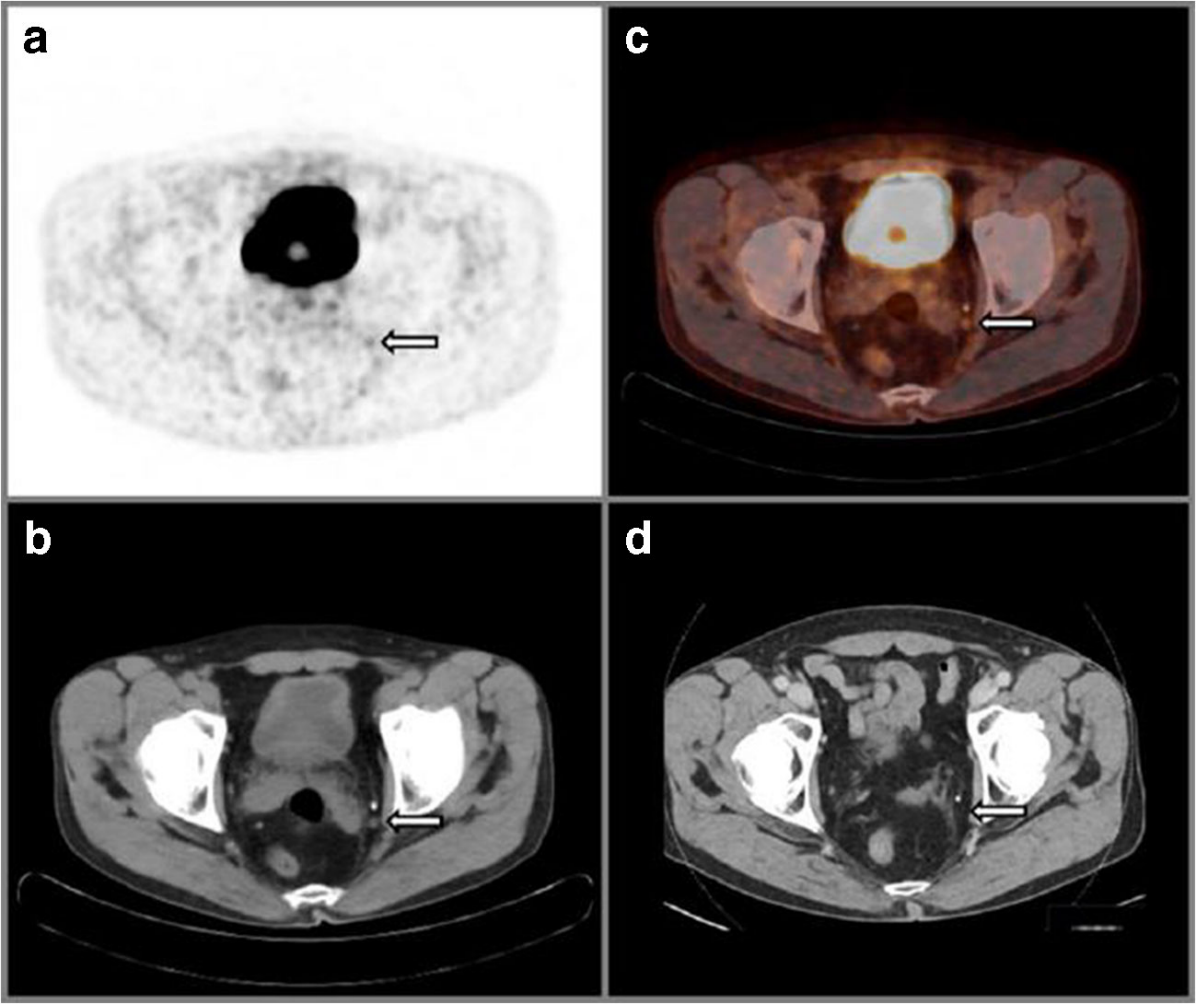
A 70-year-old man with cT2b Gleason score 4 + 5 = 9 prostate cancer and initial PSA 3.5 ng/mL considered candidate for radical prostatectomy with extended pelvic lymph-node dissection. MSKCC nomogram showed 32.0% risk of lymph-node involvement. Transversal ^18^F-DCFPyL PET (**a**) and fused PET/CT (**b**) show focal, enhanced PSMA expression in the left pelvic obturator region, compatible with a small (short axis diameter 6 mm) lymph node on CT (**c**), yet suspect for lymph-node metastasis because of increased expression in a site typical for prostate cancer, with definitive findings on CT (**a**–**c**, right arrow). After surgical resection of 26 lymph nodes, post-operative histopathology revealed no evidence of lymph-node metastases. Follow-up PSA levels 2 years after surgery remained stable at < 0.1 ng/mL, therefore making a false-positive finding very likely. Moreover, a 17 month post-operative CT scan (**d**) that was performed for the risk assessment for papillary urothelial carcinoma showed that the lymph node caudal to the known calcification was removed

The median diameter of resected lymph-node metastases was 2.5 mm (IQR 1.0–6.0). The PET/CT-detected lymph-node metastases (*n* = 12) had a median tumour size of 5.5 mm (IQR 2.4–6.6), whereas the PET/CT-undetected lymph-node metastases (*n* = 19) had a significantly smaller median tumour size of 1.5 mm (IQR 1.0–4.5) (*p* = 0.03). A clinical example of a patient with both missed and detected lymph-node metastases is shown in Fig. 3. A clinical example of a patient with a false-positive lymph-node metastasis is shown in Fig. 4.

### Local staging

**Fig. 5:**
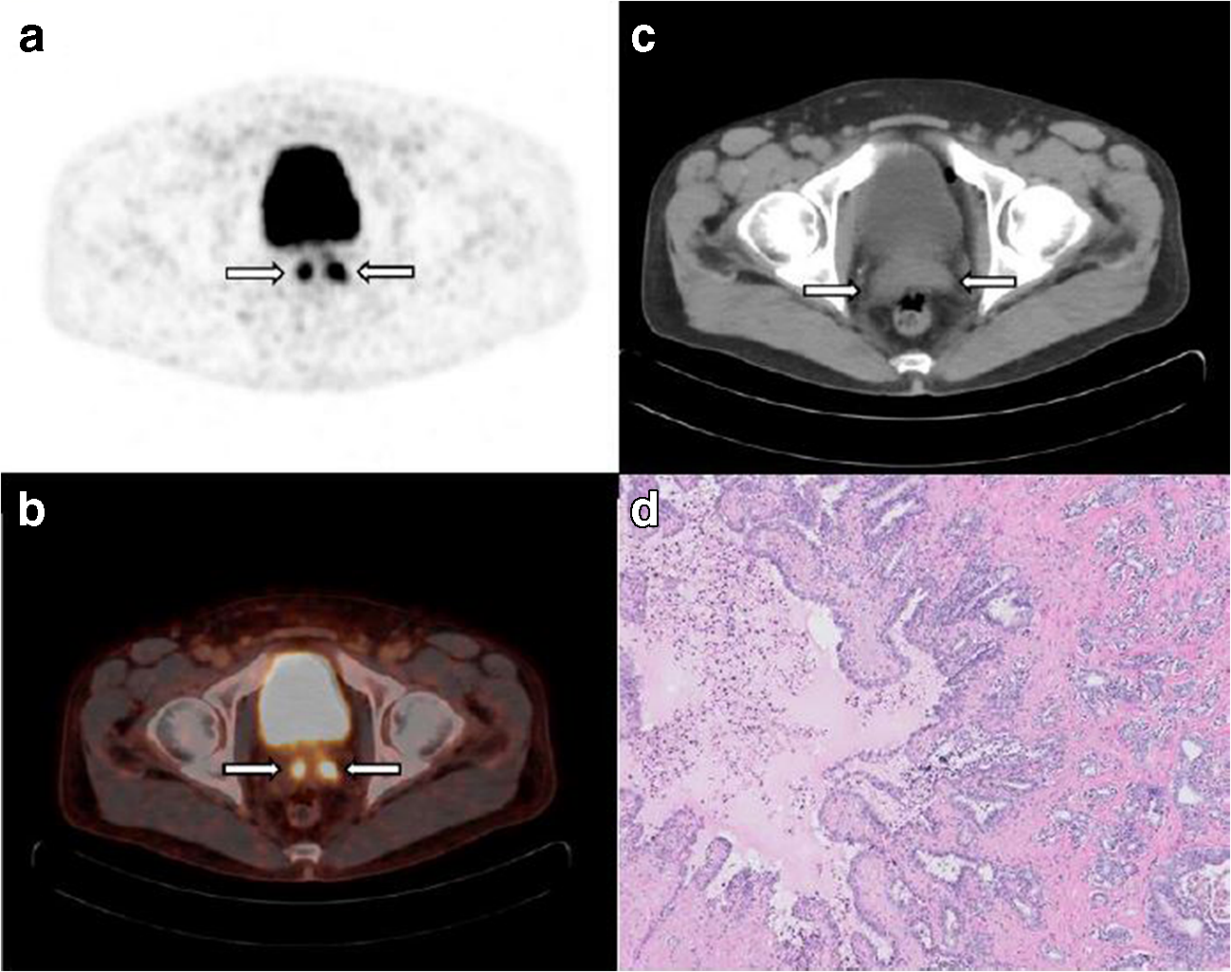
A 56-year-old man with cT2a, Gleason score 4 + 4 = 8 prostate cancer (PCa) and an initial PSA 54 ng/mL considered candidate for radical prostatectomy with extended pelvic lymph-node dissection. MSKCC nomogram showed 77.9% risk of lymph-node involvement. Transversal ^18^F-DCFPyL PET (**a**) and fused PET/CT images (**b**) revealed two foci with high PSMA expression dorsal from the urinary bladder, left and right, corresponding with bilateral seminal vesicle involvement (all arrows) on the CT (**c**). No suspicion of metastasized PCa was found using ^18^F-DCFPyL PET/CT. The primary tumour was located bilaterally in the base and midglandular regions of the prostate confirming the findings. Histopathological analysis showed a bilateral pT3b, Gleason score 4 + 5 = 9 PCa in the radical prostatectomy specimen, haematoxylin and eosin stain, original magnification × 5 (**d**). No lymph-node metastases were found after histopathological analysis of 15 resected lymph nodes

A total of 116/117 patients (99.1%) showed PSMA expression in the prostate at PET/CT. The sensitivity, specificity, PPV and NPV of ^18^F-DCFPyL PET/CT to detect locally advanced tumour growth (pT3-4) were 45.2% (95%CI 32.7–58.2%), 94.4% (95%CI 83.7–98.6%), 90.3% (95%CI 73.1–97.5%) and 60.0% (95%CI 48.8–70.7%), respectively, as seen in Table 4. For the detection of pT3a sub-stage, the sensitivity, specificity, PPV and NPV of ^18^F-DCFPyL PET/CT were 18.1% (95%CI 8.7–33.2), 97.2% (95%CI 89.4–99.5), 80.0% (95%CI 44.2–96.5) and 66.0% (95%CI 56.1–74.8), respectively. For the detection of pT3b sub-stage, the sensitivity, specificity, PPV and NPV of ^18^F-DCFPyL PET/CT were 52.9% (95%CI 28.5–76.1), 89.9% (95%CI 81.8–94.8), 47.4% (95%CI 25.2–70.5) and 91.8% (95%CI 83.9–96.1), respectively. A clinical example of seminal vesicle (pT3b) PCa involvement on PET/CT is presented in Fig. 5.

**Table 4:** The diagnostic value of ^18^F-DCFPyL PET/CT value for the prediction of local histopathologic staging (pT) after robot-assisted radical prostatectomy

pT3a-b/pT4a vs. pT2					
	pT3-4	pT2	Total	% (95%CI)	
cT3-4	28	3	31	90.3 (73.1–97.5)	PPV
cT2	34	51	85	60.0 (48.8–70.3)	NPV
Total	62	54	116	53.4	Prevalence
% (95%CI)	45.2 (32.7–58.2)	94.4 (83.7–98.6)			
	Sensitivity	Specificity			
pT3a vs. pT2/pT3b/pT4a					
	pT3a	pT2/pT3b/pT4a	Total	% (95%CI)	
cT3a	8	2	10	80.0 (44.2–96.5)	PPV
cT2/cT3b/cT4a	36	70	106	66.0 (56.1–74.8)	NPV
Total	44	72	116	37.9	Prevalence
% (95%CI)	18.1 (8.7–33.2)	97.2 (89.4–99.5)			
	Sensitivity	Specificity			
pT3b vs. pT2/pT3a/pT4a					
	pT3b	pT2/pT3a/pT4a	Total	% (95%CI)	
cT3b	9	10	19	47.4 (25.2–70.5)	PPV
cT2/cT3a/cT4a	8	89	97	91.8 (83.9–96.1)	NPV
Total	17	99	116	14.7	Prevalence
% (95%CI)	52.9 (28.5–76.1)	89.9 (81.8–94.8)			
	Sensitivity	Specificity			

*CI*, confidence interval

No significant difference in the median SUV_peak_ of the PSMA-avid prostate lesions was found between patients with and without lymph-node metastases (SUV_peak_ = 6.4, IQR 3.8–10.9, vs. 6.7, IQR 5.2–18.6) (*p* = 0.46). A Gleason score of 4 + 5 = 9 or higher was associated with a higher median SUV_peak_ when compared with the lower Gleason scores (5.8 vs. 15.0, *p* = 0.02). A Gleason score of 4 + 4 = 9 or higher was not associated with a higher median SUV_peak_ when compared with the lower scores (5.6 vs. 9.4, *p* = 0.09). No correlation was found between SUV_peak_ of the PSMA-avid prostate lesions and PSA (*R*^2^ = 0.12).

### Inter-observer agreement

Proportional agreement for the detection of lymph-node metastases using PET/CT was present in 94.7% (95%CI 89.8–97.7) overall, with a positive agreement of 78.6% (95%CI 53.4–93.9) and a negative agreement of 97.0% (95%CI 92.4–99.2). Proportional agreement for locally advanced tumours was observed in 79.1% (95%CI 71.7–85.3), with a positive agreement of 55.6% (95%CI 38.2–72.0), and a negative agreement of 86.2% (95%CI 77.9–91.5).

## Discussion

PSMA PET/CT imaging is currently the imaging technique of choice for patients with biochemically recurrent disease after initial curative local treatment (EAU guidelines) [28]. Its value for staging of primary PCa is less established, however. This the first large prospective analysis using ^18^F-DCFPyL PET/CT for primary staging of PCa, assessing the diagnostic accuracy for the detection of pelvic lymph-node metastases. The results of a total of 117 patients with intermediate and high-risk PCa that underwent ^18^F-DCFPyL PET/CT and ePLND were analysed.

In this study, ^18^F-DCFPyL PET/CT imaging demonstrated a limited sensitivity for pelvic lymph-node metastases of 41.2%, at 94.0% specificity. The limited sensitivity indicates that ^18^F-DCFPyL PET/CT does not detect all lymph-node metastases. Therefore, albeit invasive, ePLND remains the gold standard for nodal staging. The 90% NPV might suggest that a negative test is reliable in the majority of times, though we should mind the low prevalence of lymph-node metastases in this cohort (14.5%). This prevalence was congruent with the median MSKCC risk for lymph-node metastases of 14.4% (IQR 10.1–30.2). Although specificity for lymph-node metastases was favourable in this study, the PPV was moderate at 53.8% (due to the low prevalence). In this clinical setting, not all positive ^18^F-DFCPyL PET/CT results for pelvic lymph-node metastases represent actual metastatic disease. Good intra-observer agreement for the detection of pelvic lymph-node metastases using ^18^F-DFCPyL PET/CT was observed (95%).

Our results appear to be in line with a recent prospective ^68^Ga-PSMA study from van Kalmthout et al. [16]. This study used a similar methodology to our study (*n* = 103 patients), applying the same standardized ePLND techniques, histopathology analyses and PET positivity criteria. It revealed a patient-based sensitivity for lymph-node metastases of 41.5% (95%CI 26.7–57.8) and a specificity of 90.9% (95%CI 79.3–96.6) [16]. Although accuracy was similar, we should note that the prevalence of lymph-node metastases in their study was much higher (42.3% vs. our 14.5%). This is likely due to the higher proportion of patients with high-risk disease in the van Kalmthout et al. study (89.3% vs. our 63.3%). This strengthens the notion that ^18^F-DCFPyL PET/CT is at least comparable with ^68^Ga-PSMA imaging.

The high specificity presented in the current study confirms results from previous retrospective studies with ^68^Ga PSMA ligands, which reported a specificity of 90% and higher [6, 18, 19, 29, 30]. Only one prospective study reported on ^18^F-DCFPyL PET/CT as an imaging tool for initial staging of PCa [20]. In 25 patients with high-risk PCa, Gorin et al. reported a patient-based sensitivity for lymph-node metastases of 71.4% (95%CI 29.0–96.3), with a specificity of 88.9% (95%CI 65.3–98.6), at a prevalence of 28% [20]. Potentially, this higher sensitivity for lymph-node metastases is explained by the inclusion of more patients with high-risk disease (100.0% vs. our 63.3%), bearing higher PSMA expressing metastases [9].

PET/CT-detected lymph-node metastases were larger than lymph-node metastases that were not detected by PET/CT (median 5.5 mm vs. 1.5 mm, Mann-Whitney *U* test: *p* = 0.03). This may explain the imperfect imaging sensitivity reported in this study. The 5-mm spatial resolution offered by PET is still an improvement compared with the detection limits of CT and MRI (i.e. > 10 mm [4]). This discrepancy in spatial resolution could explain why PSMA PET/CT is repeatedly found to be more sensitive than conventional imaging, as confirmed by a recent meta-analysis by Wu et al. [6]. This study reported on a difference in sensitivity for the detection of lymph-node metastases of ^68^Ga-PSMA PET/CT vs. MRI (65% vs. 41%) [6].

The therapeutic consequence of PSMA-detected pelvic lymph-node metastases remains a matter of debate. Previous research showed that patients with lymph-node metastases detected intra-operatively (with frozen sections) or preoperatively (with CT) still benefit from radical prostatectomy and complete lymph-node dissection [31–34]. This suggests that detection of (a limited number of) pelvic lymph-node metastases with ^18^F-DCFPyL PET/CT should still be followed by curative treatment in the form of a RALP with an ePLND. However, the threshold to perform ePLND with certain amounts of detected number of lymph-node metastases remains unclear.

A total of 116/117 patients (99.1%) showed PSMA expression in the prostate at PET/CT. A promising PPV for the detection of pT3a-b of 90.3% (95%CI 73.1–97.5) was observed using ^18^F-DCFPyL PET/CT, yet the sensitivity was limited at 45.2% (95%CI 32.7–58.2). Moreover, the promising specificity of the detection of pT3a-b using ^18^F-DCFPyL PET/CT of 94.4% (95%CI 83.7–98.6) is in line with previous reports on ^68^Ga-PSMA (specificity > 90% for T3b) [29, 35, 36]. The high specificity for the detection of pT3a-b PCa ^18^F-DCFPyL PET/CT is comparable with that of mpMRI, for which a meta-analysis revealed a specificity of 88% (95%CI 85–97%) compared with our 94.4% (95%CI 83.7–98.6) [37]. The sensitivity, however, was better for mpMRI at 0.61 (95%CI 0.54–0.67) vs. our sensitivity of 45.2% (95%CI 32.7–58.2) [37]. Altogether, it seems that current PSMA PET/CT does not outperform mpMRI for the detection of extra-prostatic growth of PCa. Clinically, the distinction between organ confined (T2) and extra-prostatic growth (T3) is of therapeutic importance (i.e. for planning nerve sparing surgery) [3]. We recommend nuclear medicine physicians to report on the absence or presence of tracer uptake suspect for extra-prostatic growth specifically. The lower positive agreement score (56%), however, may indicate that dual reading is advisable for routine clinical care as well.

Our study has inherent limitations. Firstly, this study did not assess the accuracy of ^18^F-DCFPyL PET/CT for detecting distant metastases. Only patients undergoing RARP and ePLND were considered for analysis, which naturally excludes patients with distant metastases in which radical surgery is forgone. As such, our results should be interpreted as the accuracy of ^18^F-DCFPyL PET/CT for N-staging in patients (expected to be) free from distant metastases (i.e. eligible for radical treatment). Therefore, we decided to focus our study on determining the accuracy of ^18^F-DCFPyL PET/CT for N-staging, as hereto a solid reference standard is available (ePLND). Determining the accuracy of M-staging is certainly of interest, yet any such analysis is limited to providing a PPV, as the true prevalence of distant metastases cannot be known.

Since the PET/CT resolution is confined at 5 mm, limited diagnostic accuracy for micro metastases is to be expected. Secondly, this study might not have been adequately powered, since the expected prevalence was higher than the actual prevalence (30% vs. 14.5%). Moreover, the sensitivity used for the power analysis was higher than actually realized (90% vs. 41.2%) due to the high expectations for the sensitivity of PSMA PET/CT. Lastly, we should consider that the golden standard (ePLND) is not always flawless: in two patients with PET-positive lymph nodes, the ePLND was reported to be technically challenging. Histopathological analysis did not reveal any lymph-node metastases, yet these patients soon developed a biochemical recurrence. Repeated ^18^F-DCFPyL PET images were obtained, again detecting positive lymph nodes in the surgical template. Metastasis-directed radiotherapy to these lesions was followed by a PSA-response. The ePLND may possibly have missed these lesions initially that were rightfully detected on the first PET/CT scan.

The follow-up data of this cohort is necessary to investigate whether a specific risk profile in combination with a negative ^18^F-DCFPyL PET/CT scan could be used to withhold PCa patients from an ePLND. Future studies are therefore needed to assess whether the diagnostic accuracy of ^18^F-DCFPyL PET/CT, its high specificity in particular, could assist in proper treatment planning of patients with intermediate and high-risk stages of disease.

## Conclusion

In this prospective cohort study, we evaluated the accuracy of ^18^F-DCFPyL PET/CT imaging for the detection of lymph-node metastatic disease in men with intermediate and high-risk prostate cancer, undergoing radical surgery. We found a limited sensitivity of 41.2% (95%CI 19.4–66.5) at excellent specificity (94.0%). Based on current results, ^18^F-DCFPyL PET/CT imaging should not replace ePLND.

## Data Availability

Data are available on request to the corresponding author.
